# Unsupervised Learning by Spike Timing Dependent Plasticity in Phase Change Memory (PCM) Synapses

**DOI:** 10.3389/fnins.2016.00056

**Published:** 2016-03-08

**Authors:** Stefano Ambrogio, Nicola Ciocchini, Mario Laudato, Valerio Milo, Agostino Pirovano, Paolo Fantini, Daniele Ielmini

**Affiliations:** ^1^Dipartimento di Elettronica, Informazione e Bioingegneria, Politecnico di Milano and IU.NETMilano, Italy; ^2^Research and Development Process, Micron Semiconductor ItaliaVimercate, Italy

**Keywords:** neuromorphic circuits, spike timing dependent plasticity, phase change memory, neural network, memristor, pattern recognition, cognitive computing

## Abstract

We present a novel one-transistor/one-resistor (1T1R) synapse for neuromorphic networks, based on phase change memory (PCM) technology. The synapse is capable of spike-timing dependent plasticity (STDP), where gradual potentiation relies on set transition, namely crystallization, in the PCM, while depression is achieved via reset or amorphization of a chalcogenide active volume. STDP characteristics are demonstrated by experiments under variable initial conditions and number of pulses. Finally, we support the applicability of the 1T1R synapse for learning and recognition of visual patterns by simulations of fully connected neuromorphic networks with 2 or 3 layers with high recognition efficiency. The proposed scheme provides a feasible low-power solution for on-line unsupervised machine learning in smart reconfigurable sensors.

## Introduction

Neuromorphic engineering represents one of the most promising fields for developing new computing paradigms complementing or even replacing current Von Neumann architecture (Indiveri and Liu, 2015). Tasks such as learning and recognition of visual and auditory patterns are naturally achieved in the human brain, whereas they require a comparably long time and excessive power consumption in a digital central processor unit (CPU). To address the learning task, one approach is to manipulate the synaptic weights in a multilayer neuron architecture called perceptron, where neurons consist of CMOS analog circuits to perform spike integration and firing, while synapses serve as interneuron connections with reconfigurable weights (Suri et al., 2011; Kuzum et al., 2012; Indiveri et al., 2013; Wang et al., 2015). Recent advances in nanotechnology have provided neuromorphic engineers with new devices which allow for synaptic plasticity, such as resistive switching memory (RRAM; Waser and Aono, 2007; Jo et al., 2010; Ohno et al., 2011; Ambrogio et al., 2013; Prezioso et al., 2015), spin-transfer-torque memory (STT-RAM; Locatelli et al., 2014; Thomas et al., 2015; Vincent et al., 2015), or phase change memory (PCM; Suri et al., 2011; Bichler et al., 2012; Burr et al., 2014; Eryilmaz et al., 2014). In particular, recent works have shown the ability to train real networks for pattern learning, adopting backpropagation (Burr et al., 2014) and recurrently-connected network (Eryilmaz et al., 2014). The advantage of these devices over CMOS is the small area, enabling the high synaptic density which is required to achieve the large connectivity (i.e., ratio between synapses and neurons) and highly parallelized architecture of the human brain. In addition, nanoelectronic synapses allow for low-voltage operation in hybrid CMOS-memristive circuits, and for augmented functionality with respect to CMOS technology, thanks to the peculiar phenomena taking place in the memristive element. For instance, the CMOS-memristive synapse showed the ability to perform spike-timing dependent plasticity (STDP; Yu et al., 2011; Ambrogio et al., 2013), the transition from short-term to long-term learning (Ohno et al., 2011), a multilevel cell operation allowing for gradual weight update (Wang et al., 2015) and a stochastic operation suitable to redundant neuromorphic networks (Suri et al., 2012; Yu et al., 2013; Garbin et al., 2015; Querlioz et al., 2015).

In this context, PCM technology is an attractive solution for nanoelectronic synapse in high density neuromorphic systems. PCM is currently under consideration for stand-alone (Servalli, 2009) and embedded memories (Annunziata et al., 2009; Zuliani et al., 2013). Generally, the device appears with one-transistor/one-resistor (1T1R) architecture which allows for strong immunity to voltage variations as well as relatively compact structure. Either metal-oxide-semiconductor (MOS) or bipolar junction transistor (BJT) have been used in the 1T1R architecture. In some case, the one-diode/one-resistor (1D1R) structure has been demonstrated, capable of extremely small area and high density using the crosspoint architecture (Kau et al., 2009). The PCM technology platform has been used for computing applications for Boolean logic functions (Cassinerio et al., 2013) and arithmetic computation (Wright et al., 2011), including numerical addition, subtraction and factorization (Hosseini et al., 2015). Neuromorphic synapses have also been studied: Kuzum et al., have first demonstrated STDP in PCM by use of an ad-hoc train of pulses at either terminal of the device (Kuzum et al., 2012). Suri et al., have presented a 2-PCM synapse, where the 2 PCM devices serve as complementary potentiation and depression via gradual crystallization (Suri et al., 2011; Bichler et al., 2012). Supervised training and learning using back-propagation schemes were recently shown using PCM arrays (Burr et al., 2014; Eryilmaz et al., 2014). Despite the wealth of novel demonstrations of PCM technology, no STDP-based unsupervised learning and recognition with PCM synapse circuits has been presented so far.

Here we present a novel 1T1R synapse based on PCM capable of STDP. Potentiation of the synapse is achieved via partial crystallization enabling a gradual increase of synapse conductance, while synapse depression occurs by amorphization in the reset transition. STDP characteristics are demonstrated by experiments as a function of the initial resistance state and of the number of potentiating pulses. We demonstrate the ability to learn and recognize patterns in a fully-connected neuromorphic network and we propose for the first time the input noise as a means to depress background synapses, thus enabling on-line pattern learning, forgetting and updating. Training of the PCM synapse network with alternating and multiple visual patterns according to the MNIST data base is shown. Pattern recognition with multiple layers is finally addressed for improved learning efficiency.

## Materials and methods

### PCM characteristics

**Figure 1:**
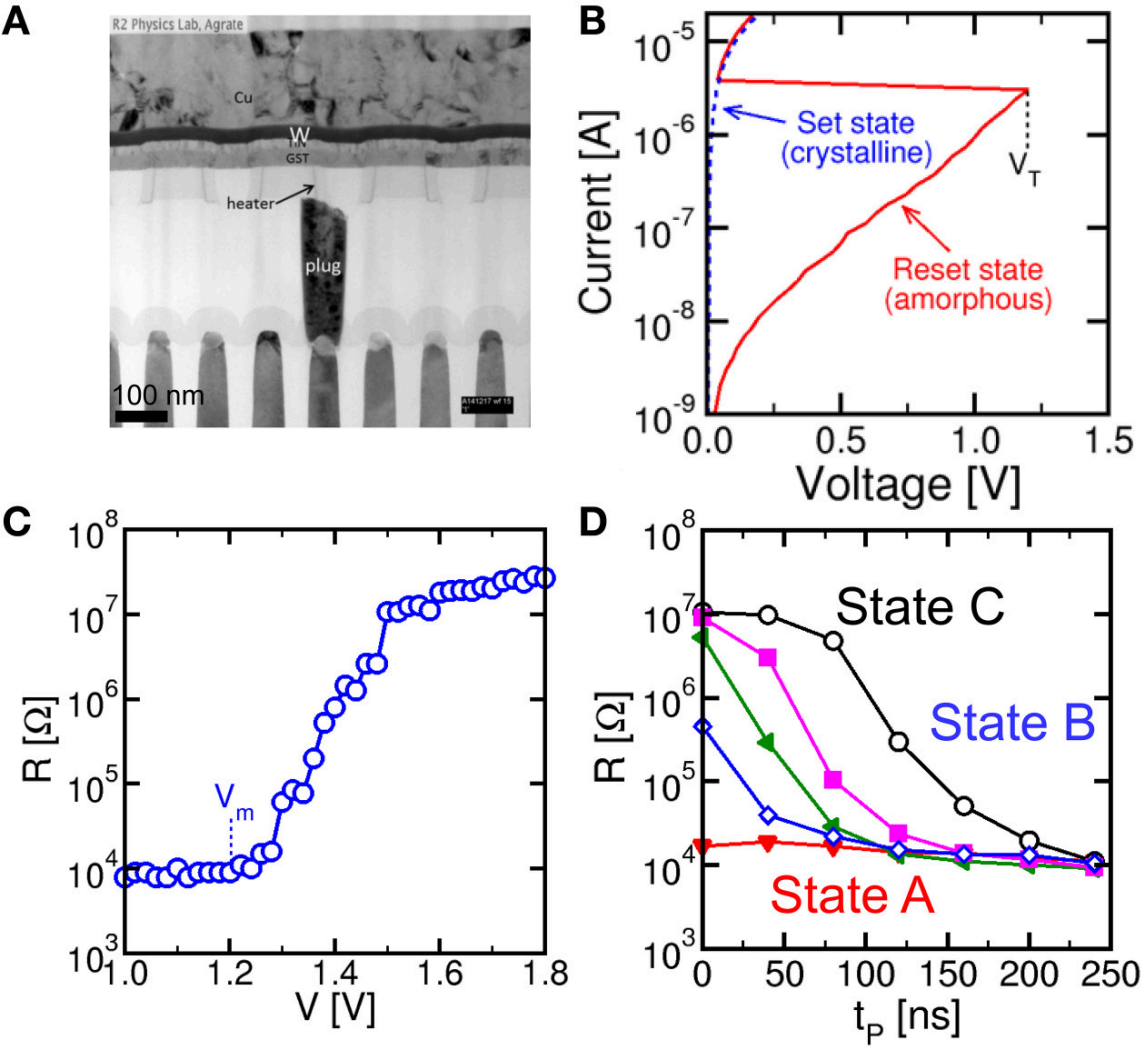
**Cross sectional view of a PCM obtained by transmission electron microscopy (TEM) (A), measured quasi-stationary I-V curves for the PCM device in the crystalline and amorphous phase (B), reset characteristic of R as a function of the write voltage for pulse-width 40 ns (C) and set characteristics of R as a function of the set pulse-width t_P_ and voltage V_set_ = 1.05 V for variable initial PCM state (D)**. The PCM device shows fast switching at low voltage, thus supporting PCM technology for low-voltage, low-power synapses in neuromorphic systems.

Figure 1 shows the PCM device used in this work (a) and its characteristics. The PCM was fabricated with 45 nm technology and consists of an active Ge_2_Sb_2_Te_5_ (GST) layer between a confined bottom electrode (or heater) and a top electrode (Servalli, 2009). The PCM top electrode was made of a Cu/W/TiN multilayer connecting all cells along a row in the array, while the bottom electrode consisted of a tungsten plug and a sub-lithographic TiN heater connected to the GST layer. The active material GST is a well-known phase change material, which remains stable in 2 phases, namely the crystalline phase and the amorphous phase (Wong et al., 2010). The 2 phases differ by their respective resistance, as displayed by the I-V characteristics in Figure 1B: while the crystalline (set) state shows a relatively low resistance, the amorphous (reset) state shows high resistance and a typical threshold switching behavior at a characteristic threshold voltage V_T_ (Ielmini and Zhang, 2007). To change the PCM state, positive voltage pulses are applied between the top electrode and the heater. Figure 1C shows the resistance R measured after the application of a rectangular write pulse as a function of the pulse amplitude V. The PCM device was initially prepared in the set state with *R* = 10 kΩ by application of a pulse with amplitude 1.2 V for 250 ns, before any applied pulse. Data show that R remains constant, until the applied voltage exceeds the voltage V_m_ for GST melting, causing amorphization, around 1.2 V, which corresponds to the melting voltage of the device. Above V_m_, the applied pulse is able to induce melting, which leaves the GST volume in an amorphous phase as the voltage pulse is completed. The amorphous volume increases with V, thus leading to the increase of R with V in the characteristic of Figure 1C. To recover the initial crystalline phase, a rectangular pulse with voltage below V_m_ is applied. A voltage V_reset_ = 1.75 V is sufficient to induce a resistance change to about 20 MΩ, corresponding to a full reset state. Figure 1D shows the resistance R measured after a set pulse with voltage V_set_ = 1.05 V as a function of the pulse-width t_P_ and for increasing initial R from 15 kΩ to 10 MΩ of the PCM (different colors in Figure 1D). In general, R decreases with increase in t_P_ as a result of the increased crystalline fraction (Cassinerio et al., 2013). A pulse width of about 250 ns is generally sufficient to complete crystallization within the GST layer irrespective of the initial value of R, thus supporting the good quality of PCM in terms of fast memory, low write voltage and low power consumption.

### 1T1R architecture

**Figure 2:**
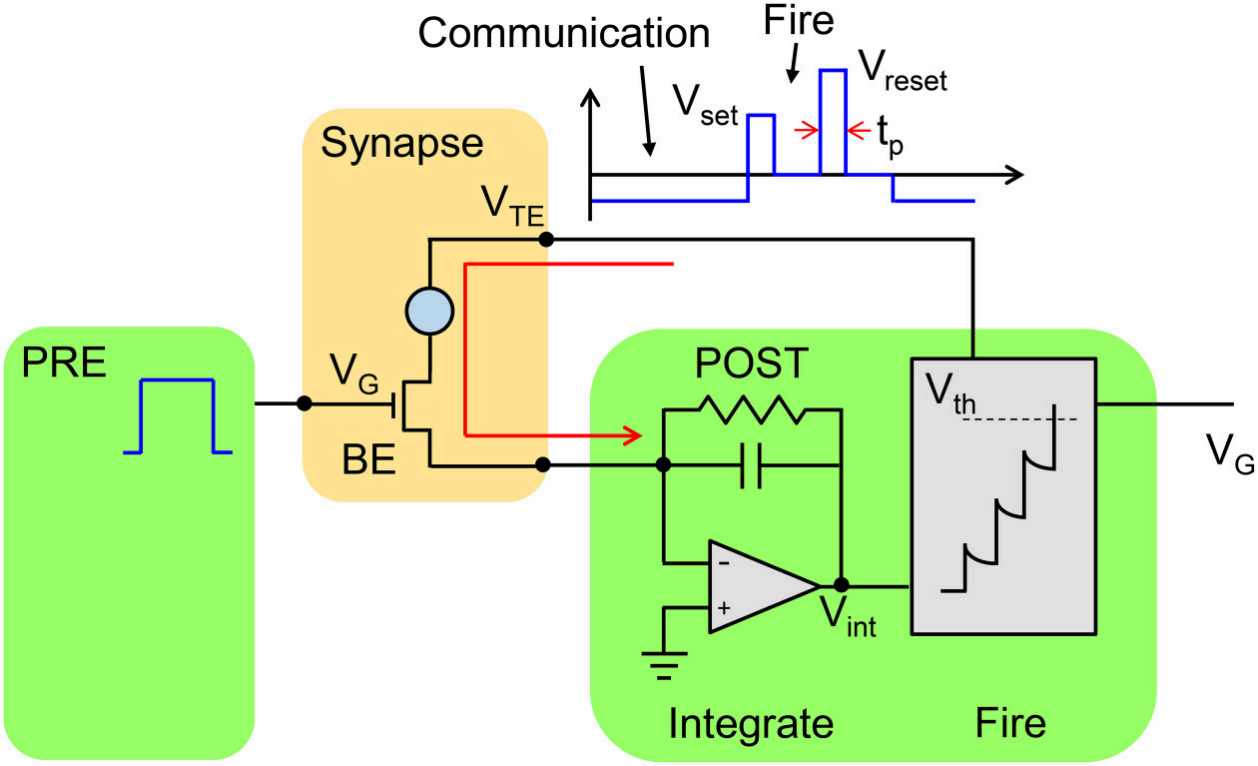
**Schematic illustration of the neuromorphic network with a 1T1R synapse**. The PRE drives the MOS transistor gate voltage V_G_, thus activating a current spike due to the low negative TE voltage (V_TE_ = −30 mV) set by the POST. The current spikes are fed into the POST, which eventually delivers a V_TE_ spike back to the synapse as the internal voltage V_int_ exceeds a threshold V_th_. The V_TE_ spike includes a set and reset pulse to induce potentiation/depression according to the STDP protocol.

Figure 2 schematically shows a neuron/synapse/neuron block of the neuromorphic network. Here, the synapse consists in a 1T1R structure where the PCM cell is connected in series with a MOS transistor. The transistor width and length must be suitable to drive a current around 300 μA, which is needed for set and reset transition in the PCM with 45 nm technology (Servalli, 2009). As a reference, an embedded PCM device with 1T1R structure has an area (almost equal to the transistor area) of 36F^2^, where F is the minimum feature size of the technology, for *F* = 90 nm and a write current of 400 μA (Annunziata et al., 2009). The 1T1R synapse has 3 terminals, namely the gate electrode of the transistor, the top electrode (TE) of the PCM and the bottom electrode consisting of the transistor channel contact not connected to the PCM. The synapse gate voltage V_G_ is driven by the pre-synaptic neuron (PRE), which applies a sequence of rectangular spikes. The positive gate voltage activates a current spike in the synapse which is fed into the post-synaptic neuron (POST). Each neuron in the neuromorphic network consists of a leaky integrate and fire (LIF) circuit, where the input current spike is integrated by the first stage, thus raising the internal (or membrane) potential V_int_. The TE voltage V_TE_ is controlled by the POST, and is normally equal to a negative constant value, e.g., −30 mV. Thanks to the negative V_TE_, a negative current spike is generated in the 1T1R in correspondence of the PRE spike, hence causing a positive increase of V_int_ in the inverting integrator of Figure 2. The relatively low V_TE_ ensures that the resistance state of the PCM is not changed, thus avoiding unwanted synaptic plasticity during the communication mode. The POST also controls the gate voltage of the synapse in the connection to the neuron in the next layer (not shown in Figure 2). Therefore, the scheme in Figure 2 represents the building block to be replicated to achieve a generic multilayer neuromorphic array. Note finally that the 1T1R synapse in Figure 2 can be considered a simplified version of the 2-transistor/1-resistor (2T1R) synapse presented by Wang et al. where communication and plasticity were achieved by 2 separate transistors (Wang et al., 2015), instead of only one transistor in the present solution.

**Figure 3:**
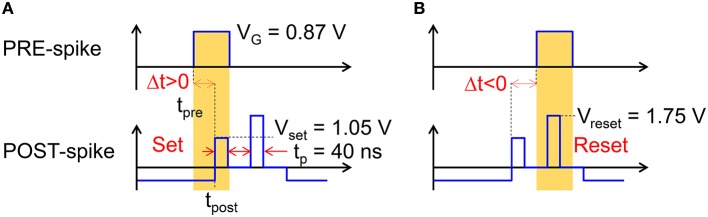
**Scheme of the applied pulses from the PRE and POST neurons to the 1T1R synapse**. In the case of small positive delay Δt **(A)**, when the PRE spike is applied just before the POST spike, the PCM receives a potentiating pulse with voltage V_set_ inducing set transition. On the other hand, for small negative delay Δt **(B)**, when the PRE spike is applied just after the POST spike, the PCM receives a depressing pulse with voltage V_reset_ inducing reset transition. For positive/negative delays larger than 10 ms, there is no overlap between PRE and POST spikes, thus no potentiation/depression can take place.

As V_int_ exceeds a given threshold V_th_ of a comparator, the fire stage delivers a pulse back to the TE to update the weight of the synapse. The TE spike contains 2 rectangular pulses, the second pulse having a higher amplitude than the first one. The specific shape of the V_TE_ spike results in a change in the PCM resistance depending on the relative time delay between the PRE and POST spikes, in agreement with the STDP protocol. STDP in the PCM synapse is illustrated in Figure 3, showing the applied pulses from the PRE and the POST. The PRE spike is rectangular, with a 10 ms pulse-width and amplitude V_G_ = 0.87 V, followed by a 10 ms after-pulse at zero voltage. The POST spike lasts 20 ms overall, and includes two pulses of width t_P_ at the beginning of the first and the second halves of the total pulse. The amplitudes of the first and second pulses are V_set_ = 1.05 V and V_reset_ = 1.75 V, respectively, intercalated by wait times at zero voltage. Amplitudes V_set_ and V_reset_ are tuned to induce set transition (crystallization) and reset transition (amorphization), respectively, according to the PCM characteristics in Figure 1. These values should be suitably adjusted according to the specific memory technology integrated in the synapse.

We define the relative time delay Δt given by:

$$\begin{array}{l} \Delta \text{t}={\text{t}}_{\text{post}}-{\text{t}}_{\text{pre}}, \end{array}$$

where t_*post*_ is the initial time of the POST spike and t_pre_ is the initial time of the PRE spike, as shown in Figure 3. If the PRE spike appears before the POST spike (a), the relative delay Δt is positive and the PRE spike overlaps with the POST spike during the set pulse of voltage V_set_, thus inducing set transition in the PCM with a consequent decrease of resistance. This corresponds to the so-called long-term potentiation (LTP) in the STDP protocol. If the PRE spike appears after the POST spike (b), the relative delay Δt is negative and the PRE spike overlaps with the POST spike during the reset pulse of voltage V_reset_, thus inducing reset transition in the PCM with a consequent increase of resistance. This corresponds to the so-called long-term depression (LTD) in the STDP protocol.

## Results

### STDP characteristics

**Figure 4:**
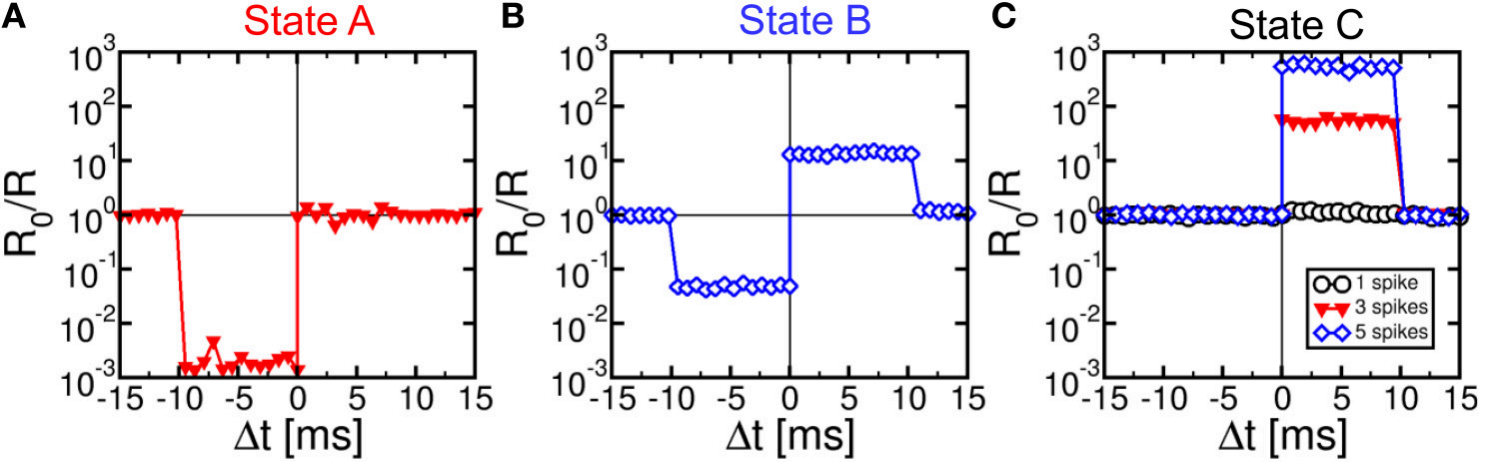
**STDP characteristics, namely measured change of conductance R_0_/R as a function of delay Δt, for various PCM states, namely state A (R_0_ = 15 kΩ), state B (R_0_ = 500 kΩ), and state C (R_0_ = 10 MΩ), also reported in Figure 1D**. Depression and/or potentiation are shown depending on delay and initial state, providing a confirmation of the STDP capability in our 1T1R synapse.

We characterized STDP characteristics in a 1T1R synapse, obtained by wire-bonding a MOS transistor and a PCM device on 2 separate chips. The transistor size was *L* = 1 μm and *W* = 10 μm and the device was able to deliver sufficient current to switch the PCM device during set and reset. To demonstrate STDP operation, voltage pulses as in Figure 3 were applied to the transistor gate and to the TE terminal with variable delay Δt and variable initial resistance R_0_ of the PCM device. We used a pulse-width t_P_ = 40 ns of set/reset pulses in the POST spike, i.e., the same as in Figures 1C,D. Figure 4 shows the measured change of conductance R_0_/R, where R_0_ and R were measured before and after the applied gate/TE pulses, for the 3 initial states of the PCM shown in Figure 1D, namely state A close to the full set state (R_0_ = 15 kΩ), state B which is intermediate between set and reset states (R_0_ = 500 kΩ), and state C close to the full reset state (R_0_ = 10 MΩ). R was measured after one spike event in all cases except for state C, where 1, 3, and 5 spikes were used in the experiments. State A (Figure 4A) displays strong depression for Δt < 0, indicating a resistance increase by about 3 orders of magnitude corresponding to the full resistance window of the PCM device between set and reset states in Figure 1C. On the other hand, state A does not show any potentiation, since the phase is already almost completely crystallized in this state. State B (Figure 4B) shows both depression (Δt < 0) and potentiation (Δt > 0), since both set and reset transition are possible for this intermediate state. Finally, state C (Figure 4C) shows no depression, since this state is already fully amorphized. In the case of one spike, the PCM also shows no potentiation, since a 40-ns pulse is not able to induce significant crystallization in the fully-amorphized state according to the set characteristics in Figure 1D. Potentiation however arises after an increasing number of spikes, reaching about a factor 10^3^× in the case of 5 repeated spikes with the same delay. These characteristics demonstrated STDP with abrupt depression and gradual potentiation due to cumulative crystallization in the PCM device (Cassinerio et al., 2013). Note that t_P_ = 40 ns was chosen to be long enough to allow for full reset of the PCM device, while providing a partial and additive crystallization according to Figure 1D. A longer t_P_ would result in slightly different STDP characteristics, due to the larger crystallization similar to the enhanced potentiation with larger number of spikes in Figure 4C. On the other hand, depression would not be affected by increasing t_P_, since the reset transition only depends on the quenching time.

**Figure 5:**
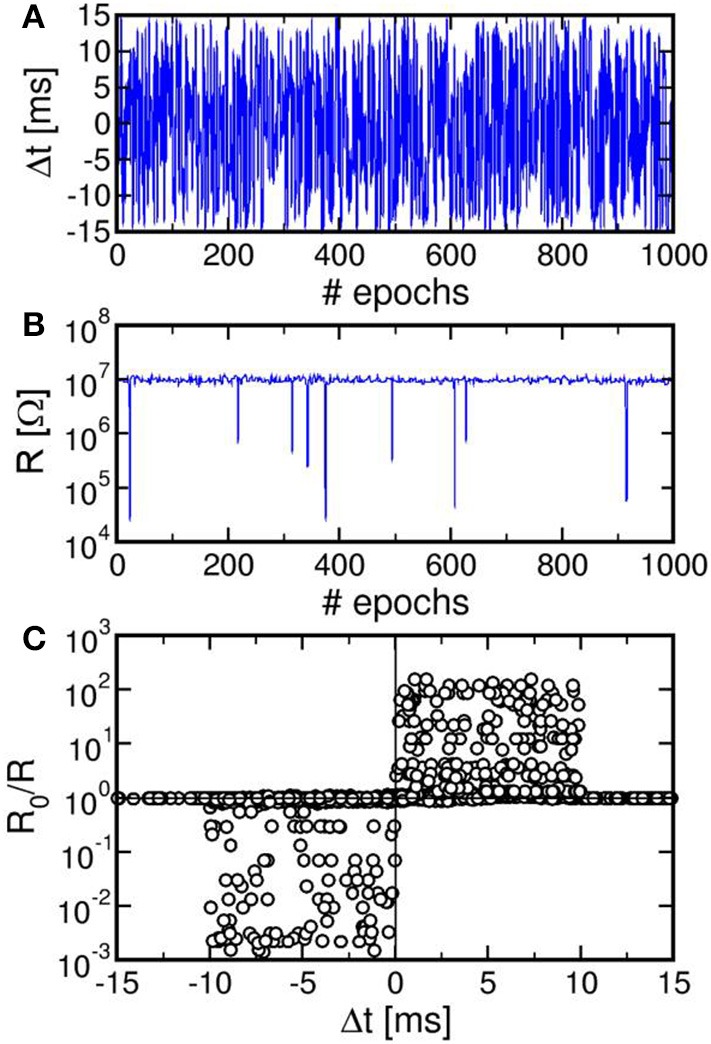
**Result of a random spiking experiment, showing the random delay Δt as a function of the epoch (A), corresponding synapse resistance as a function of the epoch (B), and correlation between Δt and R_0_/R (C)**. The correlation between delay and conductance change is consistent with the STDP characteristics at variable resistance in Figure 4.

We also verified that continuous spiking with random relative delay Δt leads to random potentiation and depression of a single PCM synapse. Figure 5 shows the results of a random Δt spiking experiment over 1000 epochs (i.e., spike events), reporting the Δt (a), the synapse resistance R as a function of the number of epochs (b), and a correlation between R_0_/R and Δt (c), where R_0_ and R were measured before and after each spike in the sequence. Due to the uniform distribution of Δt adopted in our experiment, R in Figure 5B remains close to the full reset state for most of the experiment. Only few obvious resistance drops were obtained, since at least 3 pulses with Δt > 0 are needed in Figure 4C to achieve potentiation from the full reset state. The correlation between Δt and R_0_/R over 10^4^ spikes in Figure 5C nicely agrees with the STDP characteristics in Figure 4, thus further supporting the STDP capability in our PCM–based synapse.

Note that potentiation/depression in Figures 4, 5 only take place during the set/reset pulses of pulse-width 40 ns, which is a negligible fraction of the spike timescale of 10 ms. This ensures that the energy consumption is negligible for synaptic plasticity as required by low power applications of the neuromorphic system.

### Neuromorphic network

**Figure 6:**
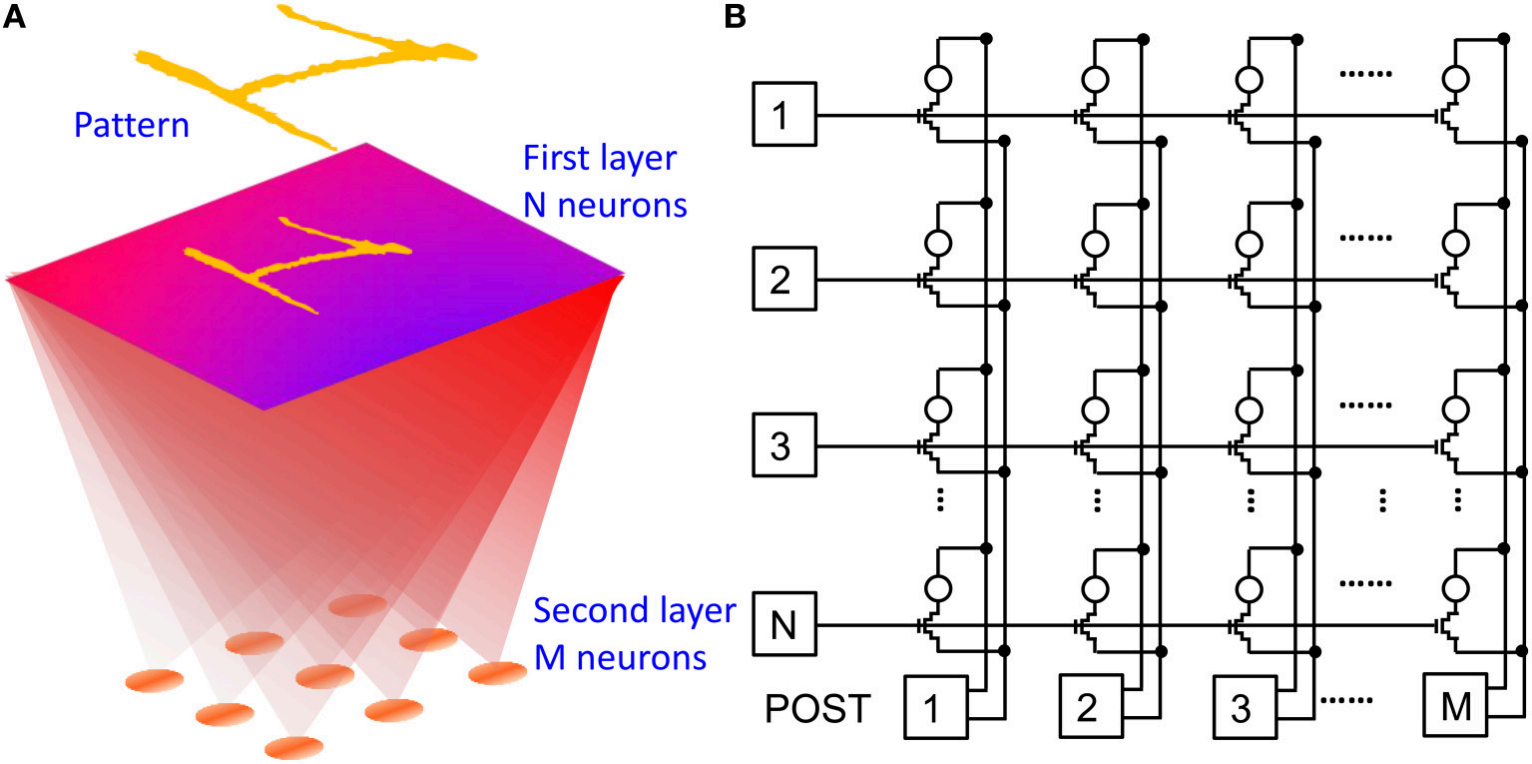
**Neuromorphic network adopted in our simulations: schematic illustration (A) and corresponding circuit (B)**. A first neuron layer with *N* = 28 × 28 neurons is fully connected to a second neuron layer with M neurons through 1T1R PCM-based synapses. The first layer delivers spikes in response to presentation of one or more visual patterns. During training, STDP within the synapses leads to LTP/LTD update of the synapse weights eventually resulting in the specialization of the output neurons in recognizing the submitted patterns.

Due to the simplicity of the POST spike shape including a set pulse and a reset pulse, the STDP characteristics in Figures 4, 5 show constant depression and potentiation for Δt < 0 and Δt >0, respectively, in contrast to the exponential-like decay which was revealed by previous *in-vivo* experiments (Bi and Poo, 1998). In addition, STDP characteristics in Figures 4, 5 are affected by a large window which can reach 1000x in one single spike, as opposed to the gradual change of only few percent of biological synapses (Bi and Poo, 1998). To demonstrate that the simplified features of our STDP do not prevent a proper learning capability in our synapse, we performed simulations of pattern learning in a fully-connected perceptron with 2 neuron layers and 1T1R PCM-based synapses. Figure 6 schematically illustrates the adopted architecture (a) and shows a practical circuit implementation with 1T1R synapses (b). The input pattern stimulates the first layer of neurons, consisting of a 28 × 28 retina in our simulations. Each of these 1st layer (PRE) neurons is connected to each 2nd-layer (POST) neurons via a synapse. We varied the number of POSTs in the 2nd layer and the intra-layer synaptic interaction depending on the purpose of the simulation. The 2-layer neuromorphic network can be arranged in the array-type synaptic architecture in Figure 6B, where a synapse in row *i* and column *j*, with *i* = 1, 2, 3, …, N and *j* = 1, 2, 3, …, M, represents the connection between the *i*-th PRE and the *j*-th POST. Therefore, the generic *i*-th PRE drives the gate terminals of all 1T1R synapses within the corresponding row, while the generic *j*-th POST receives the total current generated in the *j*-th column of synapses and drives the TE terminals of all synapses in the *j*-th column, according to the scheme in Figure 2.

### Simulation of learning of a single pattern

**Figure 7:**
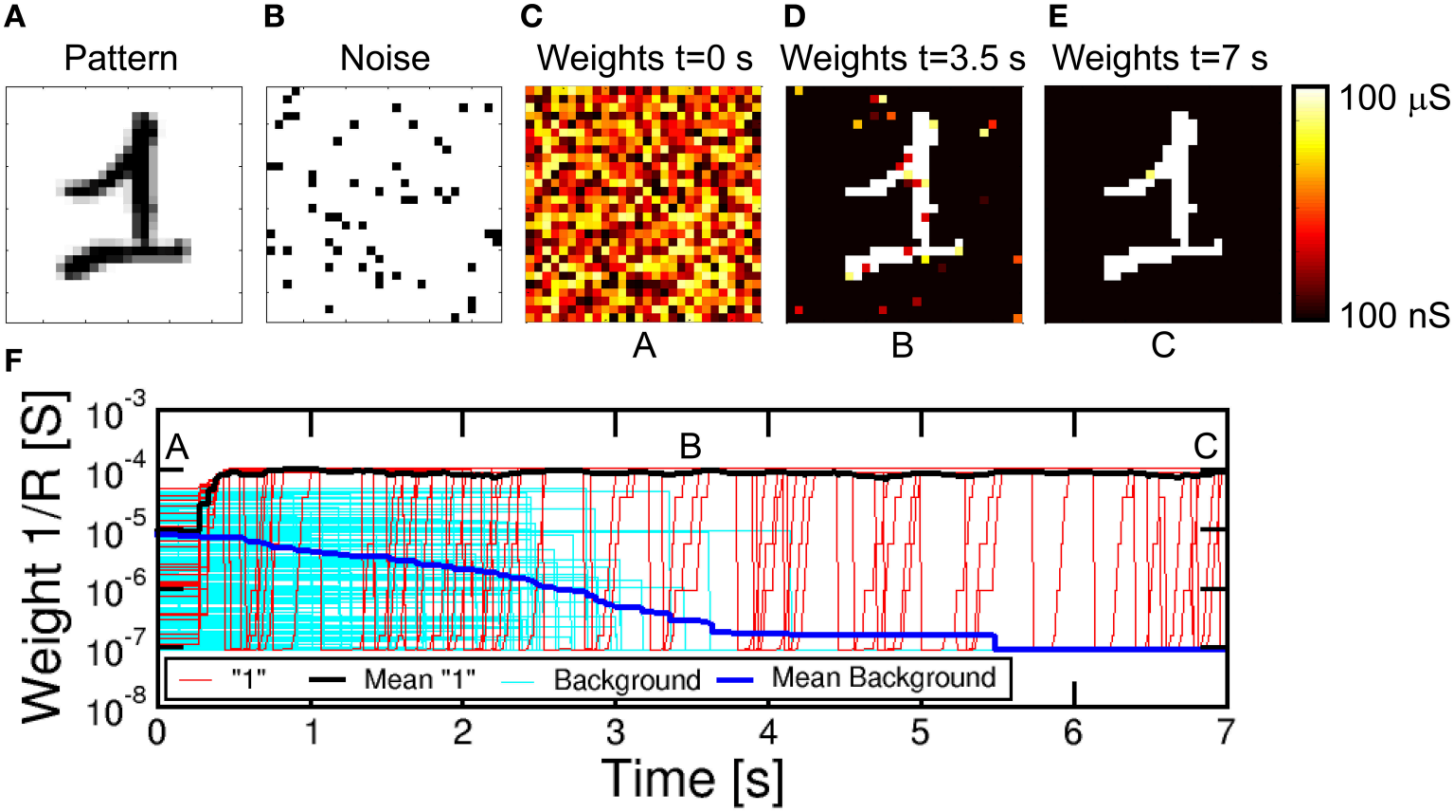
**Simulation results for pattern learning**. The input pattern “1” **(A)** is presented at the input together with noise **(B)**. Synaptic weights are random at *t* = 0 s **(C)**, then they specialize at progressive times 3.5 s **(D)** and 7 s **(E)**. The corresponding complete evolution of synapse weights for increasing time is shown in **(F)**, with positions A, B, and C related to **(C–E)**. Red lines represent synapses for pattern, cyan lines are the background synapses, while the black and blue lines are the mean pattern and background synapses, showing progressive learning and specialization.

Figure 7 shows the simulation results for the case of a 28x28 PRE retina array (*N* = 784) with a single POST (*M* = 1). Simulations were obtained with the software MATLAB and the model for PCM crystallization dynamics was obtained by interpolating data in Figure 1D. CMOS neuron circuitry was modeled with ideal integrators, comparators and arbitrary waveform generators, while the transistor in the 1T1R was modeled as a series resistance of 2.4 kΩ during communication and fire. The input pattern in Figure 7A consists of a handwritten “1” chosen within the MNIST database (LeCun et al., 1998). The pattern was randomly alternated with random noise (Figure 7B) for the purpose of inducing random spikes which uniformly depress all background synapses not belonging to the pattern. PRE-synaptic neurons were randomly activated during each noise event to allow for uniform depression of the background. Pattern and noise were presented with probability 50% each with clock time t_ck_ = 10 ms. Noise consists in the excitation of an average of 51 neurons randomly selected within the 784 PREs, corresponding to a fraction of 6.5% of neurons. During each noise epoch we extracted a different instance of white 1/0 noise. PRE spikes led to the excitation of synaptic currents that were integrated by the single POST in the 2nd layer, causing fire events every time the internal voltage exceeded V_th_.

The evolution of the synaptic weights is shown by the color maps of conductance 1/R at *t* = 0 s (Figure 7C), *t* = 3.5 s (d) and *t* = 7 s (e), also corresponding to the total simulated time. We assumed that the initial distribution of weights is random between set and reset states, which can be obtained, for instance, by initially resetting all cells, then applying relatively short set pulse with voltage close to the PCM threshold voltage V_T_. A random-set operation was shown to generate random bits in RRAM, thus enabling true random number generation (Balatti et al., 2015). Figure 7F shows the detailed time evolution of the synaptic weights, including 25, out of a total of 76, representative synapses within the pattern and other 236, from a total of 708, from the background, together with the corresponding average weights. Starting from the initial random distribution, the pattern weights (in red in Figure 7F) start to potentiate after approximately 0.3 s, reaching a value of 10^−4^ Ω^−1^ around about 0.4 s. This is the result of cumulative crystallization in the PCM as a result of multiple STDP events with Δt > 0, corresponding, e.g., to the presentation of a pattern which induces a fire in the POST. Background synapses (in cyan in Figure 7F) are instead depressed over a longer scale of about 3.5 s, where they reach a conductance of about 10^−7^ Ω^−1^ corresponding to the full reset state. The depression mechanism takes advantage of the random noise appearing at the PRE neuron layer. Since noise is uncorrelated, it only causes synapse depression when the noise PRE spike comes soon after a previous fire (thus with Δt < 0) most probably induced by pattern spikes. Therefore, noise plays a key role in depression, although it should be kept to a moderate frequency and moderate density (6.5% in Figure 7) during training to avoid interference with stable pattern learning. Note the fast pattern learning relatively to the slow background depression, as also evidenced by the evolution of synapse weights in Figure 7D at 3.5 s, where depression is still not uniformly achieved in the background. The rate of background depression might be enhanced by increasing the noise density, however at the expense of a disturbed potentiation of pattern synapses. In fact, a high noise density might lead to an increased probability of noise-induced fire, which, if followed by pattern presentation, may result in the depression of pattern synapses according to STDP. Therefore, the ideal noise density should be dictated by the tradeoff between fast background depression and efficient pattern learning. The real time evolution of synapse during a representative simulation is reported in the movie M1 in the Supplementary Material. We did not implement device-to-device variability for simplicity. However, the impact should be negligible, since the network relies on the bistable device behavior rather than on the analog weight update of the synapse (Suri et al., 2013).

### Energy and power consumption

**Figure 8:**
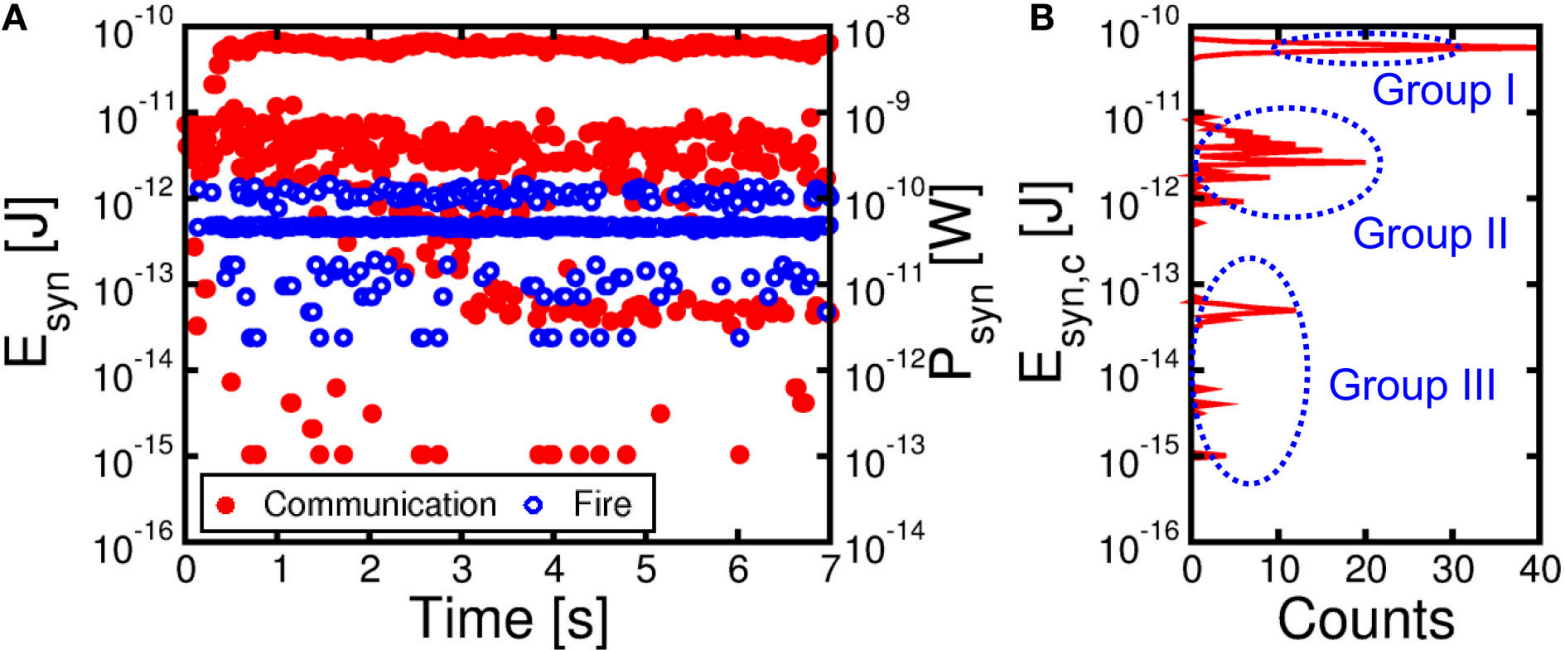
**Energy E_syn_ and mean power P_syn_ per synapse as a function of time during the learning process of Figure 7 (A) and corresponding histogram distribution of energy consumption E_syn, c_ due to communication from 4.2 s to 7 s, namely after completing potentiation/depression (B)**. Consumption due to communication (in red) is directly induced by PRE spikes, while fire energy (in blue) corresponds to set/reset events induced by POST spikes. The energy histogram reveals 3 energy levels: Group I around 80 pJ reflects communication of pattern spikes at potentiated synapses. Group II around 5 pJ represents communication of noise spikes at potentiated pattern synapses, while group III just below 100 fJ corresponds to noise spikes at depressed background synapses.

To assess the power consumption of our synaptic network, we calculated the average dissipated energy E_syn_ and power P_syn_ = E_syn_/t_ck_ per synapse, which is shown in Figure 8A as a function of time during learning. The most significant contribution to energy dissipation is due to the PRE spike (communication) which induces a current spike of t_ck_ = 10 ms due to the constant V_TE_ = −30 mV. The dissipated energy E_syn, c_ due to communication (not including fire) in a synapse is given by:

$$\begin{array}{l} {\text{E}}_{\text{syn},\text{c}}={\text{t}}_{\text{ck}}\sum_{\text{i}}{\text{V}}_{\text{TE}}^{2}∕\left({\text{R}}_{\text{i}}+{\text{R}}_{\text{MOS}}\right)∕\left(\text{NM}\right), \end{array}$$

where R_i_ is the resistance of the *i*-th synapse, R_MOS_ is the resistance of the MOS transistor in the on state, N and M are the numbers of PRE (*N* = 784 in our simulation) and POST (*M* = 1 in our simulation), respectively, and the summation is extended over all synapses that were activated by a PRE spike. In our calculations, we used a constant resistance R_MOS_ = 2.4 kΩ for simplicity. The red filled points in Figure 8A show the calculated E_syn, c_ due to the communication mode, reaching a peak of about 80 pJ as the pattern is presented to potentiated synapses after stable learning in the neuromorphic network. The corresponding dissipated power P_syn, c_ = E_syn, c_/t_ck_ is in the range of 8 nW. The dissipated energy is lower in the initial stages when the pattern is not yet learned, given the relatively low conductance of the pattern synapses.

Figure 8B shows the distribution of E_syn, c_ due to spiking communication after consolidation of weights between *t* = 4.2 s and 7 s in Figure 8A. Note that there are 3 sub-distributions of E_syn, c_, consisting of a high energy range (group I) due to pattern spiking and a low energy range, including a medium low sub-distribution (group II) and an extreme low sub-distribution (group III). Group II can be attributed to noise spikes exciting potentiated pattern synapses, which have large weights but only few are activated by the noise spikes. On the other hand, group III can be attributed to noise spikes exciting the background depressed synapses, thus corresponding to relatively few synapses with small weight on the average.

Figure 8A also shows the calculated E_syn, f_ corresponding to the fire event, when a POST spike overlaps with the PRE spike, thus giving rise to LTP or LTD. These events generally involve a much larger V_TE_ and a larger corresponding current compared to the communication spike, since updating the PCM resistance requires set and reset transitions with significant Joule heating. On the other hand, due to the short pulse-width t_P_ = 40 ns, the energy dissipation is around 1 pJ, hence negligible compared to the communication energy.

### Multiple pattern learning in sequence or in parallel

**Figure 9:**
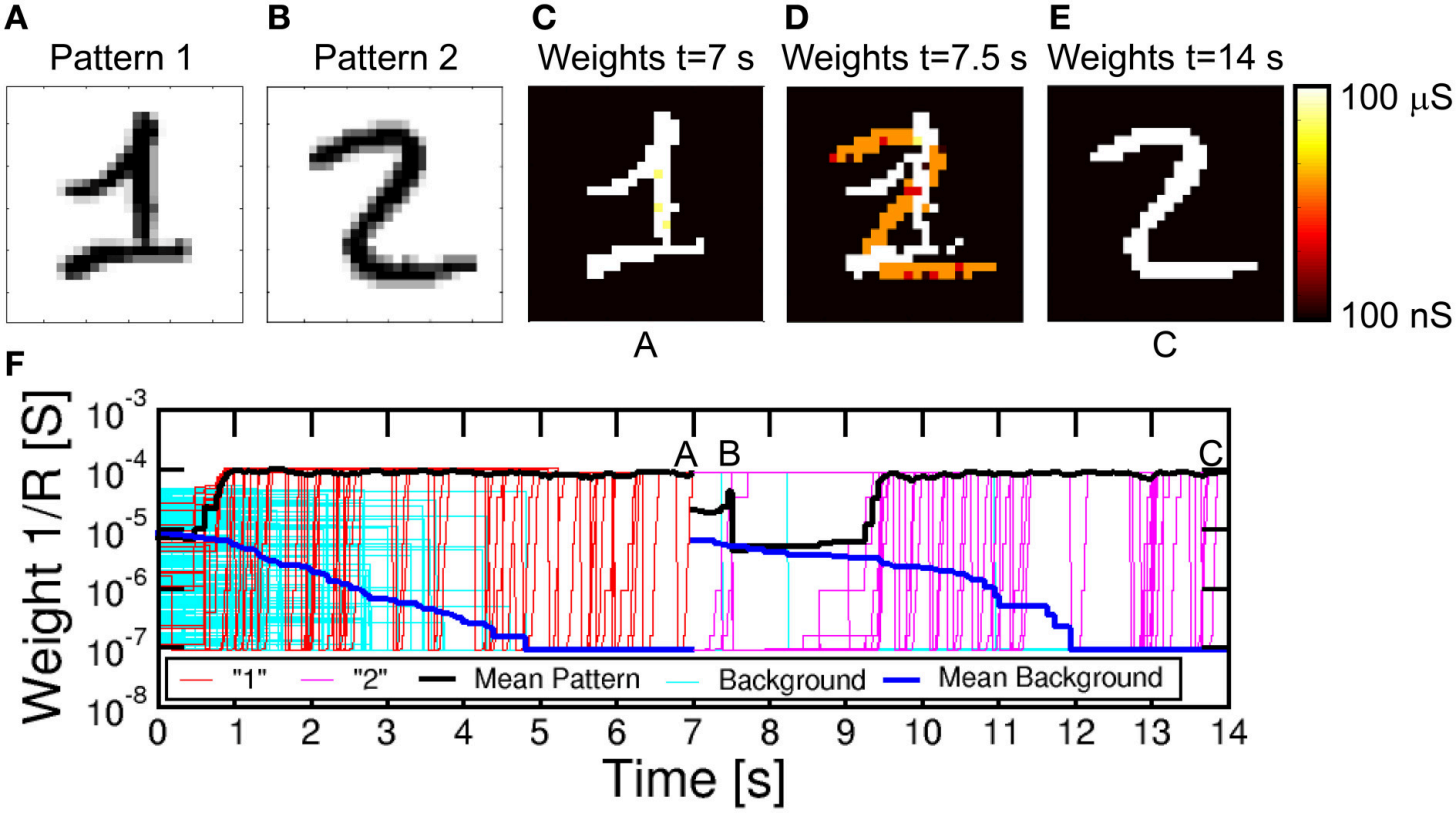
**Simulation results for pattern learning and updating. Pattern “1” and noise (A) were presented for the first 7 s, followed by pattern “2” (B) and noise for the last 7 s**. After the first 7 s, in A, pattern “1” was learnt **(C)**. After starting with “2,” synapses showed a mixed specialization at 7.5 s in B **(D)**, where “1” was being forgotten and “2” was being learned. Finally, at 14 s in C **(E)**, “2” was learnt. **(F)** shows the temporal evolution of synapses, with initial learning of “1,” followed by updating with “2.”

For on-line unsupervised pattern learning, it is important to demonstrate not only learning of a specific pattern, but also the capability to forget a previous pattern and learn a new one. The ability to reconfigure synaptic weights by learning a new pattern is in fact a key feature to rapidly interact with stimuli from a continuously-changing environment as in the real world. To verify the reconfiguration function in our neuromorphic network, we presented an input pattern to the PRE neurons for 7 s, then we presented a different pattern, where both the first and second patterns were chosen from the MNIST database. Figure 9 shows the simulation results, including the first pattern (a), the second pattern (b), the color maps of the synaptic weights for *t* = 7 s (c), *t* = 7.5 s (d), and *t* = 14 s (e), and the synaptic conductance 1/R as a function of time (f). During the initial 7 s, pattern “1” and noise were provided with equal probabilities of 50%: the average synaptic weights show a potentiation of pattern synapse weights at 0.5 s, which is in line with Figure 7. At the same time, the background synapses are gradually depressed and the pattern is completely learnt after 1 s, as also shown by the weights at 7 s in Figure 9C. After 7 s, the input pattern is suddenly changed from “1” to “2,” which causes depression of weights within pattern “1” and potentiation of weights in pattern “2.” No conductance change is seen for synapses remaining in the background or pattern area. Pattern “2” is fully learned around 9 s, with depression taking slightly longer time. Sequential learning of 2 patterns is further described by movie M2 in the Supplementary Material.

**Figure 10:**
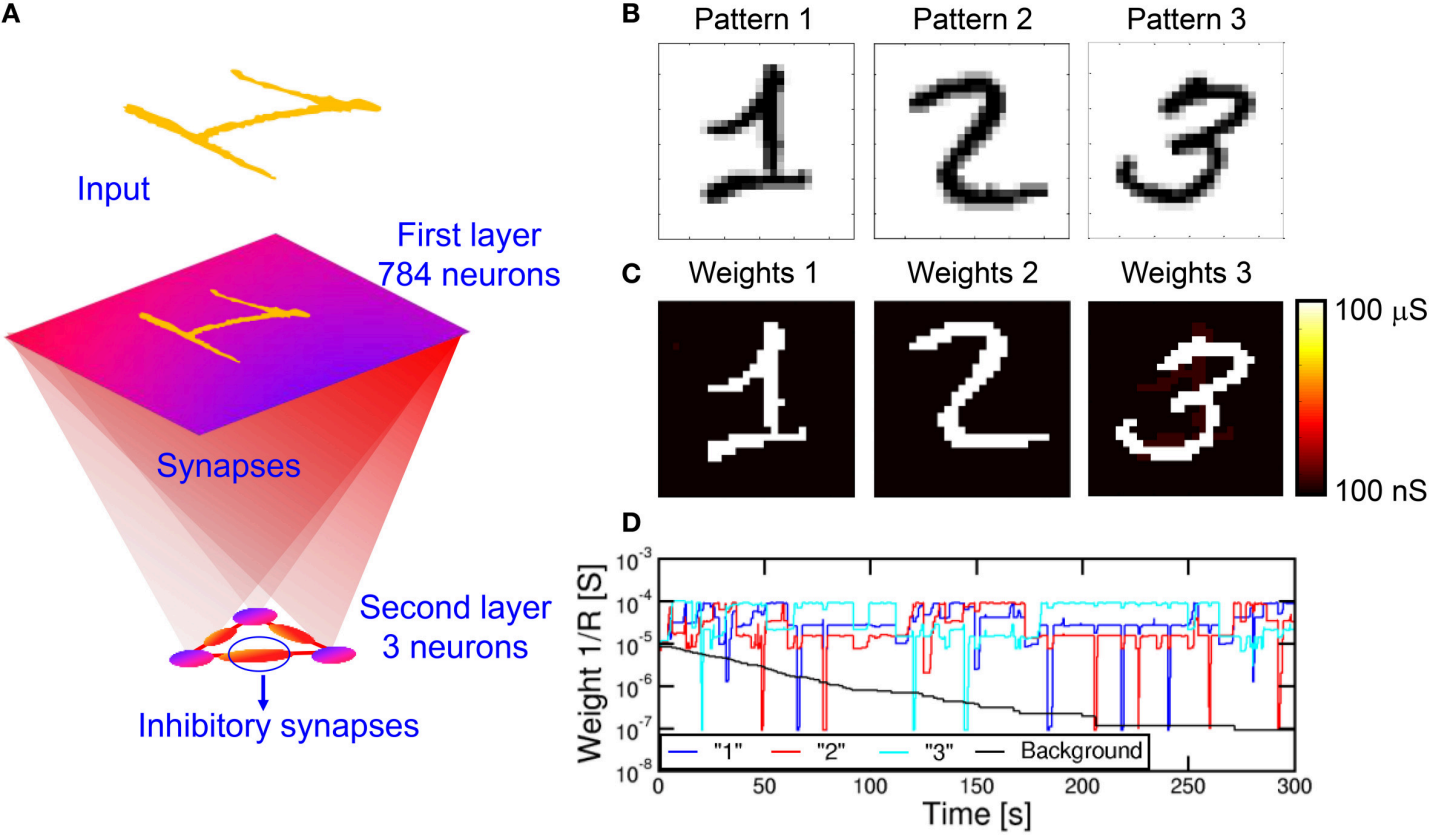
**Simulation results for multiple pattern learning**. A first layer with 28 × 28 = 784 neurons is fully connected to three second layer neurons, each of them connected with three inhibitory synapses **(A)**. We provided three patterns “1,” “2,” and “3” **(B)** to the input. The three neurons specialize on different patterns **(C)**. **(D)** shows the evolution of the synapses connected to one of the post neurons, in particular the mean weight for synapses of pattern “1,” “2,” “3” and background. While the background gradually decreases, the learnt pattern (the highest mean conductivity) changes during time due to interference between patterns.

We also verified the capability to learn multiple patterns in parallel, rather than in sequence as in Figure 9. Since a neuron can only specialize to one pattern at a time (see Figure 9), we extended the simulation to a network of multiple M neurons in the POST layer. Figure 10A shows a fully connected network including N PRE neurons and 3 POST neurons in the 2nd layer, where 3 different patterns were presented alternatively as shown in Figure 10B. The purpose is that each of the 3 neurons eventually specializes to a separate pattern, thus emulating the capability to recognize different patterns, such as letters, numbers, or words, by our brain. To avoid co-specialization to the same pattern, the 3 neurons were connected by inhibitory synapses, where a successful fire in any neuron leads to a partial discharge of the internal potential in all other neurons, to inhibit fire in correspondence of the same pattern and encourage specialization to other patterns. The inhibitory synapses have fixed weights, hence they can be implemented by simple resistors. The 3 input patterns in Figure 10B were presented with 5% probability each, with the remaining 85% consisting of noise with an average number of PRE spikes of 4 per epoch, or 0.5% of all PREs. Such low percentage of noise activity over PREs is balanced by a relatively large frequency of noise equal to 85%. After a simulated total time of 300 s, the 3 different patterns were learnt each in a different neuron, as shown by the final synaptic weights in Figure 10C. Decreasing the pattern presentation rate below 5% in Figure 10 would result in a lower learning rate, while increasing the rate would cause learning instabilities. We have observed, in fact, that high pattern presentation rates cause the network to learn superposed patterns (e.g., a “1” plus a “2”) or difference patterns (e.g., a “1” with the pixels of “2” excluded). This results from interaction of distinct patterns in the STDP. A low pattern rate helps reducing the probability of having interaction between different patterns.

Figure 10D shows the synaptic weights as a function of time, including the pattern weights and background weights (only synapses belonging to the background in all 3 patterns were shown). Learning takes place in a relatively short time at the beginning of the simulation, while depression of background weights requires about 200 s due to the low activity of noise. Note also the significant oscillations of pattern weights, which are due to the instability of pattern weights due to noise. In particular, the neuron specializes on one single pattern at a time, corresponding to the highest conductance of 10^−4^Ω^−1^. However, the network is unable to stabilize on a single pattern due to the interference with different patterns. Nonetheless, the network is able to recognize distinct patterns in distinct POST neurons, although sometimes different POSTs learn the same input pattern. This is an unwanted effect due to the low inhibitory effect we used in the simulations, where we discharged only 20% of the capacitance of a neuron during the inhibitory action. The increase of the inhibitory factor would improve the selectivity to input patterns, although it would also cause the blockade of some POST neurons due to repeated fire in another successful POST neurons. In summary, a careful trade-off must be searched to minimize blockade events, maximize the learning efficiency and minimize the learning time. Parallel learning of 3 patterns is further described by movie M3 in the Supplementary Material.

## Discussion

### Reducing power consumption via spiking communication

**Figure 11:**
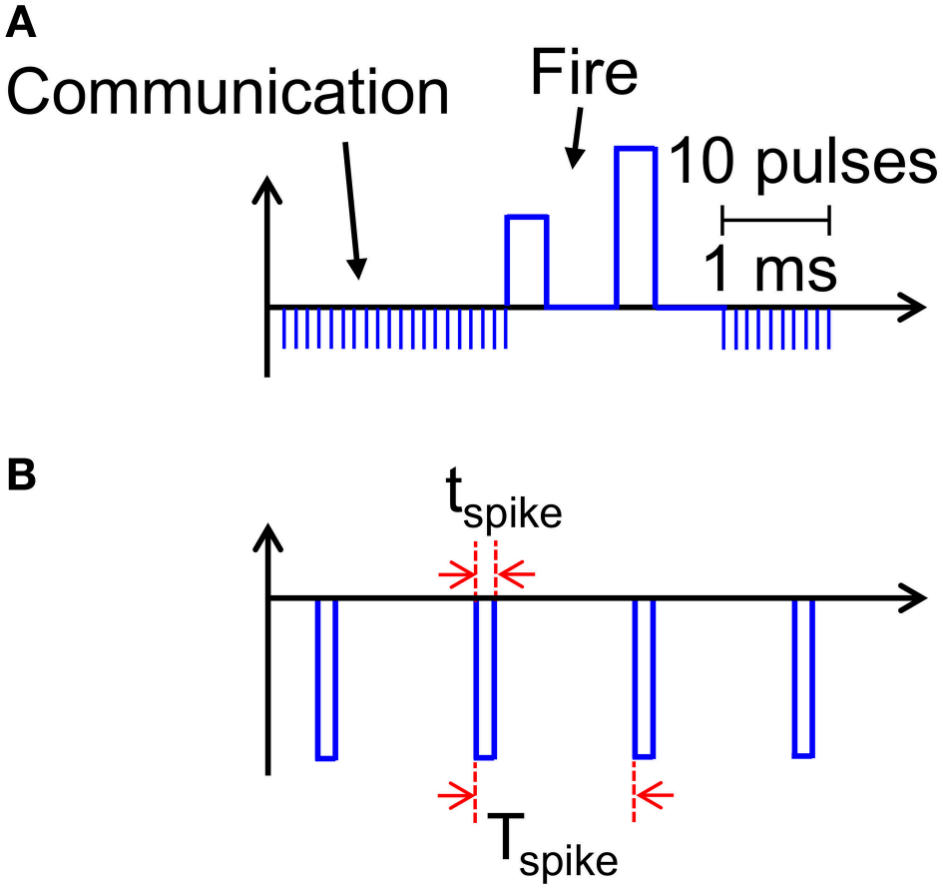
**Scheme for implementing low energy consumption communication**. Instead of applying a constant V_TE_ = −30 mV, sequences of spikes lasting t_spike_ can allow for efficient communication **(A)**, while reducing energy and power consumption by a factor t_spike_/T_spike_, where T_spike_ is the time between adjacent pulses **(B)**.

Our results support PCM devices as highly-functional synapses with learning capability and low power consumption required for the synaptic plasticity. A key limitation of the proposed scheme is however the relatively large power consumed during communication (Figure 8). Assuming a synapse density of 10^11^ cm^−2^ as in the human cortex, a power per synapse of 8 nW would translate in a power density of almost 1 kWcm^−2^, which is comparable to a multicore CPU in conventional Von Neumann computing. The large power consumption is due to the relatively long current spike lasting 10 ms in response to the PRE spike applied to the transistor gate, where the relatively long pulse width is dictated by the STDP dynamics in the 10–100 ms time scale for real time learning and interaction (Bi and Poo, 1998). However, a spiking V_TE_ can be adopted to reduce the dissipated energy during the spike. For instance, Figure 11 shows a spiking waveform of V_TE_, consisting of pulses of t_spike_ = 1 μs width and spiking period T_spike_ = 1 ms, corresponding to a spiking frequency of 1 kHz and a duty cycle of 10^−3^. The reduced duty cycle results in a reduction of power consumption by a factor 10^3^, clearly bringing our neuromorphic solution in the territory of low power chips.

An additional advantage of adopting a spiking V_TE_ with low duty cycle is the ability to reduce the capacitance in the neuron integrator stage. In fact, the capacitance can be estimated by:

$$\begin{array}{l} \text{C}\approx \Delta \text{Q}∕{\text{V}}_{\text{th}}, \end{array}$$

where ΔQ is the integrated charge contributed by the current, equal to ΔQ = IΔt in the case of a constant V_TE_ as in Figure 2. Assuming an array of 784 PRE neurons with 10% potentiated synapses after learning, a V_TE_ of −30 mV, a resistance of potentiated synapse of 15 kΩ, and a comparator threshold voltage V_th_ = 0.5 V, we obtain a capacitance of about 3 μF, which is clearly unfeasible in an integrated circuit. A duty cycle of 10^−3^ would result in a reduction of the capacitance by a factor 10^3^, hence in the range of few nF. Further reduction of the power consumption and of the integrator capacitance can be obtained by reducing the duty cycle, the value of V_TE_, and the conductivity of the PCM in the potentiated state, e.g., by adopting suitable low-conductivity phase change materials or by reducing the size of the heater controlling the cross section of the PCM device. Separation of communication and fire paths by 2T1R architecture of the synapse would allow to further reduce the current consumption and capacitor area by adopting sub-threshold bias and short pulse width of the communication gate (Kim et al., 2015; Wang et al., 2015). Finally, adopting accelerated, non-biological dynamics of tenths of ns instead of 10 ms range could allow for smaller values of integrated capacitances in the range of hundreds of fF.

Another issue consists in the wire capacitance charging energy, which is higher in the pulsing scheme. Synapses are arranged in a relatively large array, hence wires would cause a high parasitic capacitance, leading to an increase in capacitive energy dissipation in the pulsing scheme. One way to reduce the issue is to arrange synapses in a multiple smaller synapse arrays, with shorter interconnects. This approach would reduce the fan-in/fan-out of the neurons, however, with a proper design of the neuromorphic network, the issue could be acceptable, while preserving the reduction in the energy dissipation due to synapses. The capacitive energy would also be reduced by suitable voltage scaling via PCM engineering.

### Multi-layer neuromorphic network

**Figure 12:**
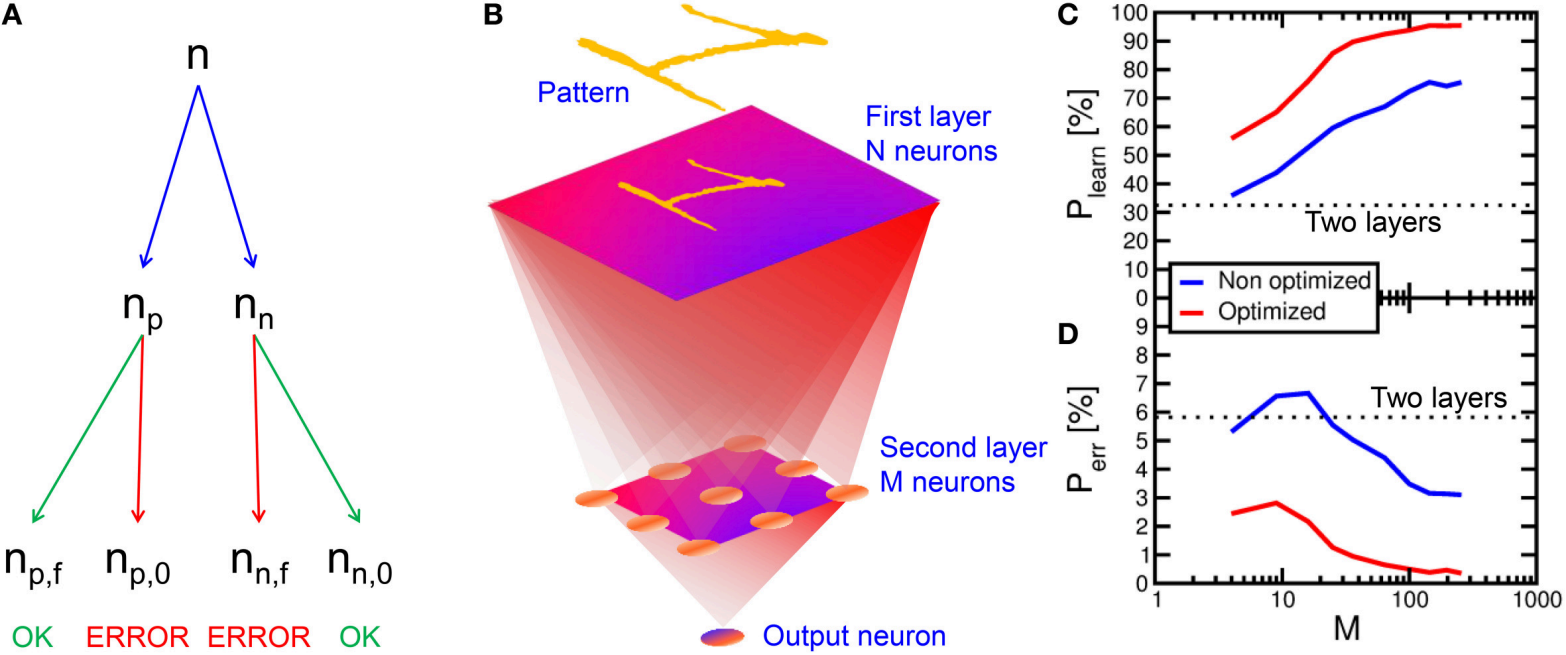
**Multi-layer simulation results**. The number n of PRE spikes is composed by n_p_ pattern and n_n_ noise inputs. n_p_ is composed by n_p, f_ (pattern leading to output spike) and n_p, 0_ (missing recognition). n_n_ is composed by n_n, f_ (false recognition) and n_n, 0_ (absence of spike for input noise) **(A)**. After an input layer with 28 × 28 neurons, a second layer with variable M neurons and a third layer with one output neuron are implemented **(B)**. The recognition rate P_learn_ = n_p, f_/n_p_ increases with respect to the two layers network and it increases for increasing number M of second layer neurons **(C)**, while the error rate P_err_ = n_n, f_/n_n_ decreases **(D)**. P_learn_ further increases for optimized conditions (lower noise), reaching a 95.5% recognition, while P_err_ drops to 0.35%.

To assess the learning efficiency of the neuromorphic network with PCM synapses, we performed 100 simulations of pattern learning with a total time of 2 s per each simulation. We evaluated the recognition probability P_learn_ as the number n_p, f_ of fire events in the POST neuron in correspondence of the presentation of pattern “1,” divided by the total number n_p_ of appearances of the same pattern, P_learn_ = n_p, f_/n_p_ (see Figure 12A). Similarly, we evaluated the error probability P_err_ as the number n_n, f_ of POST fire events taking place in correspondence of the presentation of noise in the input (false recognitions) divided by the total number n_n_ of input noise appearances, P_err_ = n_n, f_/n_n_. Note that n_p_ + n_n_ = n, where n is the total number of PRE spikes within the 2 s interval of simulation. With a 2-layer network with 28 × 28 PREs and 1 POST neuron, P_learn_ was equal to 33% and P_err_ was around 6%, thus quite unsatisfactory for the purpose of on-line learning and recognition. We found that unsuccessful learning was due most of the times to depression events of pattern synapses in the case of noise causing a POST fire, followed by the presentation of the pattern in the input. In fact, PCM is particularly prone to complete depression for Δt < 0, since the reset pulse results in a large resistance increase in just one shot. After this depression event, potentiation of pattern synapses is quite difficult, since the current flowing in the depressed pattern synapse is extremely low, making a POST fire event in response to the presentation pattern quite unlikely.

To solve this issue and improve the recognition probability, we implemented a 3-layer network, as sketched in Figure 12B. This was done by inserting an intermediate layer with M neurons between a 28 × 28 input retina and an output layer consisting of a single neuron. All neurons between the first and the second layer were connected, and all second-layer neurons were connected to the output neuron, making the network a fully-connected architecture. The number M of neurons in the second layer was varied to study the recognition efficiency and error rates with the same pattern and noise conditions as in the calculations in Figure 7. Figure 12 shows the calculated recognition probability (c) and the error probability (d) as a function of M. The recognition probability increases with M from almost 36% up to 76%, while the error rate decreases from 6 to 3%, as shown by the blue lines. The improvement is due to the compensation of synapse blockade by the additional layer, thanks to the increased number of parallel channels.

To further improve the network efficiency, we reduced the input noise from 6.5 to 5.5%. The optimized results are shown by the red curve in Figures 12C,D. The noise reduction leads to a slight increase in the time needed for depression of background synapses. On the other hand, the recognition efficiency increases up to 95.5% for 256 neurons in the second layer, while the error probability decreases to 0.35% in a 2 s simulation time. These results strongly support PCM-based neuromorphic chip for on-line unsupervised learning and recognition.

### Impact of noise density on learning efficiency

**Figure 13:**
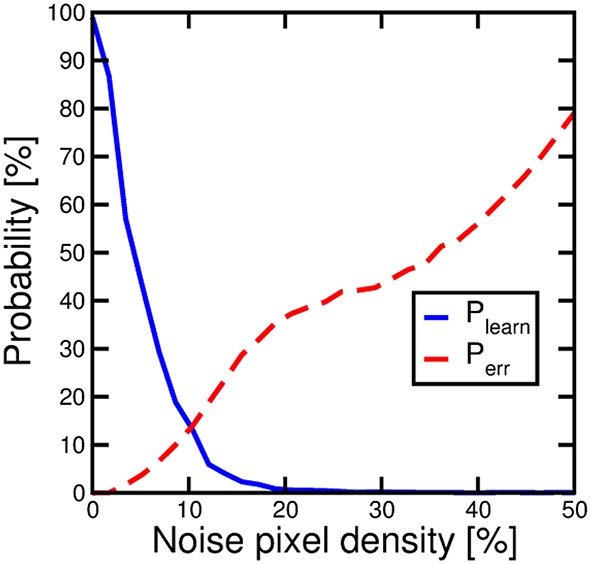
**Probability of recognizing an input pattern P_learn_, solid line, and probability of spurious fires P_err_, dashed line, as a function of input noise**.

Noise presentation alternated to the pattern allows for proper background depression and on-line unsupervised pattern updating. The randomness and non-correlation of noise allow for a general background depression and, in general, a forgetting mechanism. Figure 13 explores more deeply the impact of noise on learning efficiency. We performed pattern learning simulations as in Figure 7, varying the input noise density, namely the average percentage of PRE delivering a noise spike. P_learn_ shows a decrease for increasing noise density which is explained by the competition between pattern learning caused by pattern input appearance and increasing pattern forgetting induced by noise. At the same time, for increasing noise, P_err_ increases due to the increasing noise current contribution. However, note that zero noise, which seems to be the best situation, is not applicable, since background depression and pattern updating as in Figure 9 would not be possible. Therefore, a careful trade-off between noise density and learning performance must be considered.

In conclusion, our work demonstrates PCM-based electronic synapses based on 1T1R architecture. The synapses are capable of STDP thanks to the time-dependent overlap among PRE and POST spikes in the 1T1R circuit. On-line pattern learning, recognition, forgetting and updating is demonstrated by simulations assuming the alternation of pattern and noise spikes from the PRE layer. Reduction of energy consumption and improvement of recognition efficiency are discussed with the help of simulation results. These results support PCM as promising element for electronic synapses in future neuromorphic hardware.

## Author contributions

SA provided simulations of neuromorphic circuits for learning and recognition, while NC and ML contributed experimental data. All authors discussed the results and contributed to manuscript preapration. DI supervised the research.

### Conflict of interest statement

The authors declare that the research was conducted in the absence of any commercial or financial relationships that could be construed as a potential conflict of interest.
